# Supplementary material for: The interactome of intact mitochondria by cross-linking mass spectrometry provides evidence for coexisting respiratory supercomplexes
Source: Mol Cell Proteomics. 2017 Dec 8;17(2):216–32. doi: 10.1074/mcp.RA117.000470 (PMC5795388; doi:10.1074/mcp.RA117.000470)
Supplement: Supplemental Data [file supp_RA117.000470_133922_0_supp_23978_fzffwf.zip › spectra_annotation/mito_spectra_annotation/213-1-3-1-7-1.pdf]

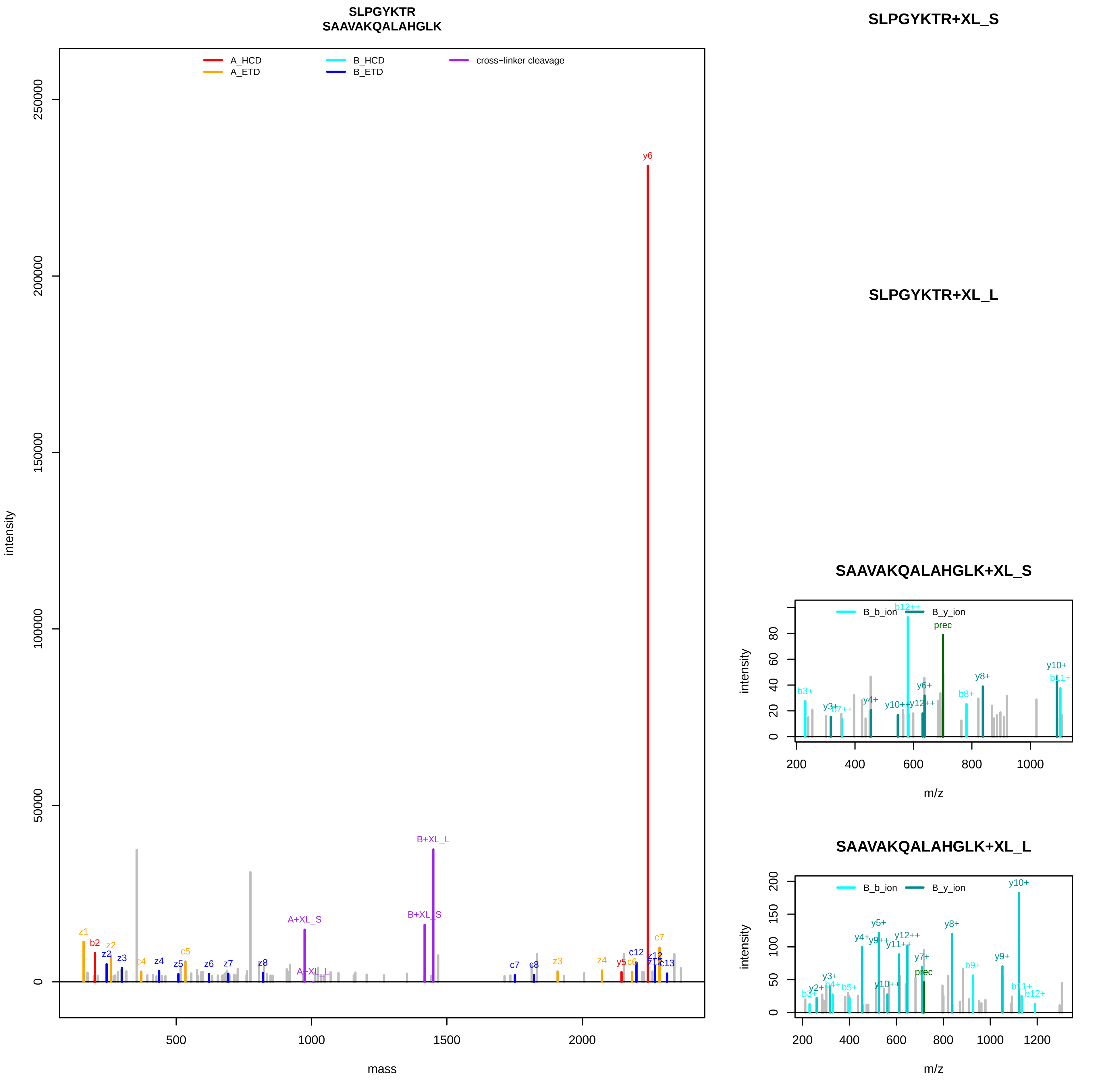
SLPGYKTR
SAAVAKQALAHGLK
A_HCD
A_ETD
B_HCD
B_ETD
cross−linker cleavage
intensity
mass
0
50000
100000
150000
200000
250000
500
1000
1500
2000
y6
B+XL_L
B+XL_S
A+XL_S
A+XL_L
z1
b2
z2
z3
c4
z4
c5
z5
z6
z7
z8
c7
c8
z3
z4
y5
c6
c12
c7
z12
c13
SLPGYKTR+XL_S
SLPGYKTR+XL_L
SAAVAKQALAHGLK+XL_S
B_b_ion
B_y_ion
b12++
prec
y10+
b11+
y8+
y6+
b8+
b3+
y3+
b7++
y4+
y10++
y12++
intensity
m/z
0
20
40
60
80
200
400
600
800
1000
SAAVAKQALAHGLK+XL_L
B_b_ion
B_y_ion
y10+
y8+
y5+
y4+
y9++
y12++
y11++
y7+
prec
b9+
y9+
y3+
y2+
b4+
b5+
y10++
b3+
b11+
b12+
intensity
m/z
0
50
100
150
200
200
400
600
800
1000
1200
